# An accessible scheme for monitoring free‐roaming cat population trends

**DOI:** 10.1002/ece3.5982

**Published:** 2020-01-21

**Authors:** Idit Gunther, Lior Azriel, Hila Wolf, Tal Raz, Eyal Klement

**Affiliations:** ^1^ Koret School of Veterinary Medicine, The Robert H. Smith Faculty of Agriculture, Food & Environment The Hebrew University of Jerusalem Israel

**Keywords:** fertility control, free‐roaming cats, population control, population monitoring, sterilization, trap–neuter–return

## Abstract

Free‐roaming cats (FRCs) form nondomiciliary population groups that might lead to adverse environmental effects, as well as to welfare impairment of the cats themselves. Though criticized by ecologists, for the last two decades, the trap–neuter–return (TNR) programs were often employed aiming to manage these populations. At present, no accepted and accessible monitoring scheme exists to determine the effectiveness of those programs. In the current study, we present the reliability and validity of an applicable monitoring scheme, as an adjunct tool for a TNR program of FRC in an urban environment. The monitoring scheme is based on cat observation counts along randomly chosen transects. Fifty‐four transects were repeatedly walked for three years, between 2012‐2014, in 27 neighborhoods within an urban area of 19.3 Km^2^. Cat numbers counted in the 2014 observations were significantly higher than cat numbers found in the 2012 observations (prevalence ratio = 1.258, CI_95%_= 1.198–1.322, *p* < 0.001). The method revealed high reliability when different observers and different transects in the same neighborhood were compared (*R* ^2^ = 0.548 and *R* ^2^ = 0.391, respectively, for measuring cat counts per km, *p* < 0.001; and *R* ^2^ = 0.5 and *R* ^2^ = 0.74, respectively, for measuring neutering percentage, *p* < 0.001). This scheme was constructively validated by measurements of municipal data on the number of neutered cats and demonstrated high correlation (*R* ^2^ = 0.59, *p* < 0.001). Conducting cat observations using friendly calling and feeding resulted in an increased number of FRC observed per km walk (by 79% and 22%–30%, respectively). However, these manipulations did not alter the recorded percentage of neutered cats. The proposed scheme provides spatio‐temporal data that can contribute to the management programs of such cat metapopulations in an urban environment.

## Graphical abstract

The presentation of the reliability and validity of an applicable monitoring scheme of free‐roaming cats in an urban environment.

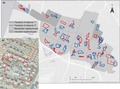

**Abbreviations**
FRC: free‐roaming cats
TNR: trap–neuter–return

## 1 INTRODUCTION

Individuals of certain domesticated species around the globe have become nondomiciliary and have formed human‐independent (or partially independent) population groups. One of the most prominent examples of this phenomenon is that of free‐roaming cat populations, as was reported in the United States (Calhoon & Haspel, 1989; Kilgour et al., 2017; Schmidt, Pierce, & Lopez, 2007); in Italy (Natoli et al., 2006); in Japan (Izawa, Doi, & Ono, 1991); in Israel (Finkler, Hatna, & Terkel, 2011; Mirmovitch, 1995); in France (Kaeuffer, Pontier, Devillard, & Perrin, 2004); and in South Africa (Jones & Downs, 2011). Such unrestricted populations can cause adverse ecological effects (Loss, Will, & Marra, 2013; Moseby, Peacock, & Read, 2015) as well as constitute a hazard to public health (Gerhold & Jessup, 2013; Gunther, Raz, Berke, & Klement, 2015; Morters et al., 2013). Moreover, the welfare of these animals is commonly impaired (Gunther et al., 2015; Gunther, Raz, & Klement, 2018; Nutter, Levine, & Stoskopf, 2004). Consequently, in the last few decades, efforts have been made to control the overpopulation of free‐roaming cats, employing culling or fertility‐control programs. Despite being criticized by ecologists, claiming its ineffectiveness for diminishing FRC numbers and their related ecological adverse effects (Lepczyk et al., 2010; Longcore, Rich, & Sullivan, 2009; Loss & Marra, 2018; Peterson, Hartis, Rodriguez, Green, & Lepczyk, 2012), the trap–neuter–return (TNR) has become one of the most commonly applied fertility‐control methods (Boone et al., 2019; Denny & Dickman, 2010; Longcore et al., 2009).

A valid estimate of free‐roaming cat (FRC) population size and dynamics is essential for planning and monitoring the effectiveness of any control strategy for these unrestricted populations (Greenwood & Robinson, 2006; Thompson, White, & Gowan, 1998). To date, however, systematically collected data on FRC populations are scarce (Boone & Slater, 2014). A review of the literature reveals that the few studies in which such data have been collected relate to one of the following three aspects:

1. Studies primarily aimed at estimating population size. These have been mostly designed as cross‐sectional, short‐term studies, employing accepted population sampling methods (e.g., capture–mark–recapture and distance sampling). Such studies have been used for size estimation of FRC populations (Calhoon & Haspel, 1989; Finkler et al., 2011; Flockhart, Norris, & Coe, 2016; Kilgour et al., 2017; Schmidt et al., 2007) and free‐roaming dog (FRD) populations (Hiby et al., 2011; Punjabi, Athreya, & Linnell, 2012) in urban settings. For long‐term use and in highly dense populations, these methods require intensive professional effort and may suffer from interference in their fundamental assumptions. Such interference in the capture–mark–recapture method can result from loss of marks, due to high population turnover (Belo, Werneck, Silva, Barbosa, & Struchiner, 2015; Gunther, Finkler, & Terkel, 2011; Kilgour et al., 2017), as well as from uneven probabilities of capturing different individuals due to differences in their habituation to humans. Interference in the distance sampling method can result from obstructed visibility; obstacles on side roads/alleyways that limit strait line transects to main roads only; an FRC density that is not constant but in patches; and a highly heterogenic architecture in the same neighborhood, necessitating multiple sampling.
2. Studies aimed at examining behavior, individual characteristics, and home range of FRCs, with present population size constituting a secondary outcome. These studies have been mostly designed for small‐sized local populations and conducted over a longer period of time, using census surveys (complete enumeration) of individually recognized FRC (Devillard, Say, & Pontier, 2003, Izawa et al., 1991, Mirmovitch, 1995, Natoli, 1985, Page, Ross, & Bennet, 1992; Say et al., 1999). The estimated densities were calculated simply by dividing the estimated number of FRC by the total area. Such studies are clearly impractical to conduct in a large area such as an entire neighborhood or city.
3. Studies aimed at describing and monitoring the proportion dynamics of neutered animals in population management programs, in order to estimate the population size using a mark–resight survey methodology. To the best of our knowledge, the sole example of such a study exists only for FRD (Hiby et al., 2011).

Monitoring population size, composition, and dynamics does not necessarily require the determination of absolute FRC numbers, but it does require a reliable and consistent estimate of changes in population size and composition. Since monitoring population management programs require a long‐term follow‐up of the controlled populations, there is a need for a reliable, valid, and feasible monitoring scheme. Lack of technical guidelines regarding the implementation of such schemes contributes to the difficulties in determining the effectiveness of management programs, as well as clouding the debate on this issue among the scientific community and in the public arena (Boone & Slater, 2014).

Free‐roaming cat management and monitoring programs are often conducted by local authorities, government officers, and animal rights organizations, and not by professional ecologists (Galvis et al., 2015, Gunther et al., 2015; Hughes, Slater, & Haller, 2002, Kreisler, Cornell, & Levy, 2019, Natoli et al., 2006, Zito, Aguilar, Vigeant, & Dale, 2018). Consequently, within this reality, there is a necessity to develop a monitoring scheme that is both reliable and valid on the one hand, and simple and applicable on the other hand. In the current study, we propose a simple monitoring scheme for the collection of data to be used for estimating population trends over time. The scheme is based on the performance of cat observation counts along randomly chosen transects. The overall objective of this study was to assess the reliability and validity of the proposed scheme, using a mixed study design, combining repeated cross‐sectional FRC surveys with a cohort study of municipal records in two cities in Israel during 2012–2014 and in 2016. Accordingly, our specific objectives were as follows: (1) to compare three approaches to observing FRC; (2) to determine the degree of interobserver agreement; (3) to determine the reliability of the sampling frame; and (4) to determine the constructive validity of this scheme through comparison with municipal records data.

## 2 MATERIALS AND METHODS

### 2.1 Study population

The 2012–2014 study was conducted in the city of Rishon LeZion, Israel. The city population comprised 237,600 citizens at the end of 2013 (Central Bureau of Statistics, Israel), living in a jurisdiction area of 50 km^2^. Rishon LeZion is located within the greater Tel Aviv metropolis and is divided into 28 residential neighborhoods, three commercial and industrial areas, and one area of research institutes. One of the industrial zones and the area of research institutes, being secluded areas and poorly occupied by human activity, therefore differ substantially from the other areas of the city and were excluded from the analysis. Additionally, four smaller neighborhoods were merged with their surrounding larger neighborhoods, resulting in a final analysis of 25 residential neighborhoods and two industrial zones.

The municipal veterinary services of the city have been carrying out a multi‐annual TNR (trap–neuter–return) program since 2009 (Table 1). During the performance of this program, the veterinary services requested owners of pet cats that roam outdoors to mark their cats with collars. FRCs that had undergone ovariohysterectomy or castration procedures were marked by cutting their ear tip. The marking procedure was performed under general anesthesia during the sterilization procedure (Cuffe, Eachus, Jackson, Neville, & Remfry, 1983). Following recovery, the FRCs were released back at the same location where they had been trapped. The municipal veterinary services kept meticulous records for each neutered FRC, including the date and the location of trapping (documented as the street address closest to the trapping location).

**Table 1:** Annual neutering numbers of FRCs, sterilized as part of a municipal TNR program in the city of Rishon LeZion, Israel

Year	Number of neutered cats
2009	434
2010	2778
2011	2360
2012	2314
2013	1564
2014	1351

Determining the effect of using friendly vocalization (calls) to draw out the cats, on the sampled percentage of neutered cats, was conducted during 2016 in the adjacent city of Rehovot, at a time when the percentage of neutered cats in the city of Rishon LeZion was very high (ca. 80%, according to counts conducted prior to this part of the study in random locations in most of the city's neighborhoods). Therefore, a preliminary pilot study was performed in the city of Rehovot, in which ca. 30% of FRC were estimated to have been neutered. The human population of Rehovot comprised 135,726 citizens at the end of 2016 (Central Bureau of Statistics, Israel), living in a jurisdiction area of 23.72 km^2^.

### 2.2 Data collection

**Figure 1:**
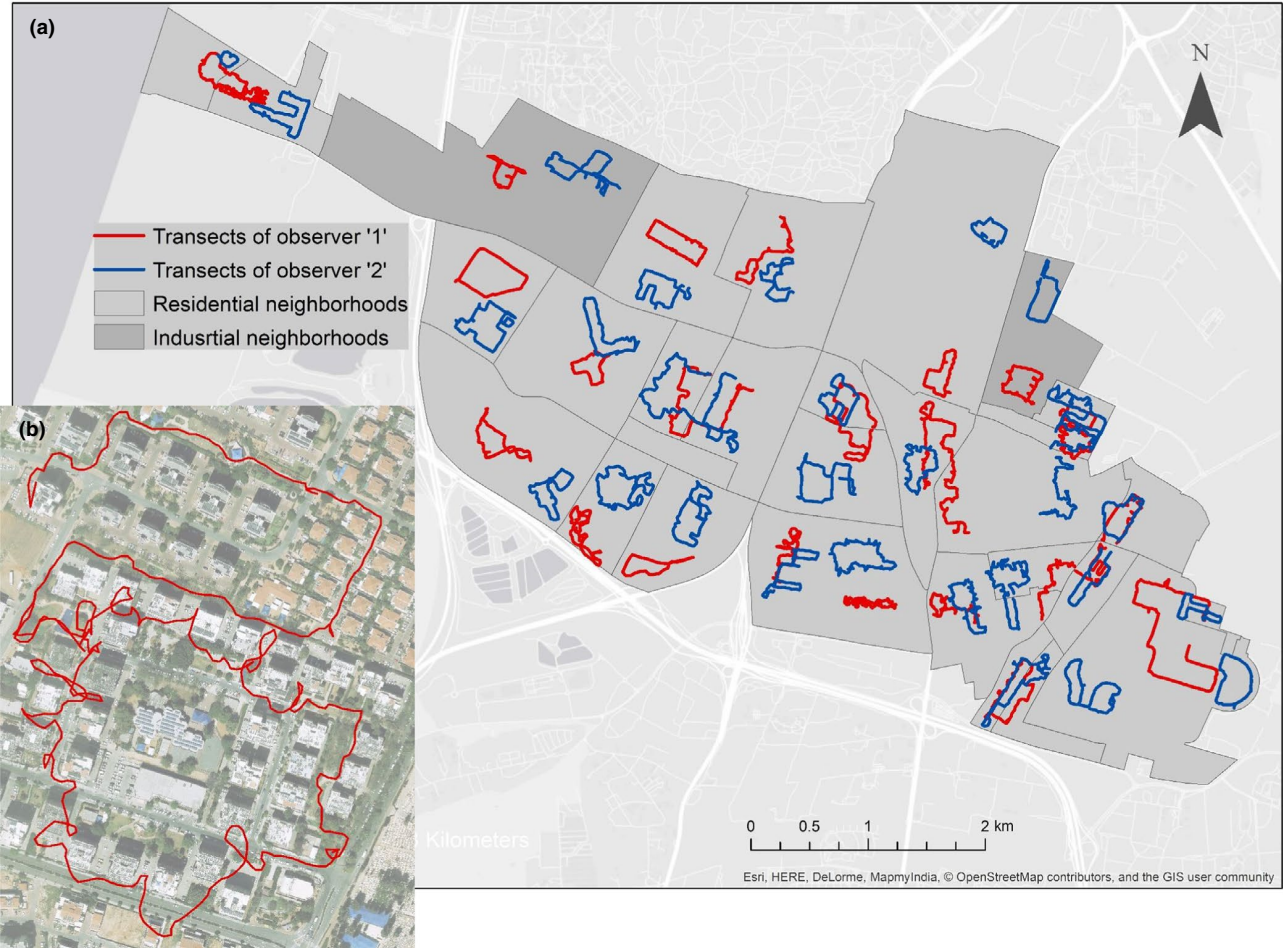
A map of the city of Rishon LeZion showing cat sample survey transects. (a) Two sets of randomly chosen observation transects (red lines, overall length of 46.79 km and blue lines, overall length of 54.08 km) in each of the 27 neighborhoods of the city. Minimum length of each transect in each neighborhood was one kilometer. Observations were performed twice a year during September and October between 2012 and 2014 by two observers. (b) An example of one enlarged transect

The study design comprised repeated surveys of stratified random samples. A sample unit (transect) was randomly chosen from a stratified geographical area (neighborhood) and was censused annually between 2012 and 2014, each September and October. During these two months of observations, TNR actions were discontinued by the municipality. Transect selection was accomplished by two observers in the first year of the study (2012), by choosing a random starting point in each neighborhood and walking randomly for between 1 and 2.5 km, relative to the area of the neighborhood. For a more thorough observation of the adjacent ten‐meter area on either side of the transect path, transects included walking in courtyards and parks that were accessible to the public. The total length of the first observer's set of transects was 46.79 km and that of the second observer's set was 54.08 km (Figure 1). To ensure walking on precisely the same path of each transect, the transect walks were recorded using a cellular GPS recording application (“Endomondo™—Running & Walking, Android application,” Under Armour Inc., Maryland, USA).

In each transect, for each observed cat we recorded its contraceptive status, as either neutered (marked by the cut tip of the ear) or intact (not marked); estimated age status, as either kitten (up to 6 months) or adult (above 6 months); and sex, determined systematically only for adults through the presence of testes in sexually intact males, the scrotum in neutered males, bulky cheeks in males, enlarged mammary glands of lactating queens, and the overall body size (males are usually larger than females).

Observations were performed from a distance without physically handling the cats. Therefore, in order to discern the external physical details of the cats, these observations were conducted during the daytime between 6:00 and 9:00 a.m. We sought to document each cat only once in each walk, regardless of the number of times they were observed. Since visibility in the urban setting is limited due to the wealth of hiding places for cats, the cats were encouraged to reveal themselves by calling them, using friendly vocalization, and by food delivery. To test the assumption that food delivery would improve the cats' visible presence, we walked each transect twice. In the first walk, cats were drawn out only by means of friendly vocalization; and in the second walk, they were drawn out using both vocalization and food delivery. The same dry commercial food (Friskies™, Purina®) was used in all sampled areas, and these two walks were conducted in each transect within a time interval of 1–4 weeks, during 2012 and 2014.

In order to determine the degree of interobserver agreement between the two observers, in 2013 two walks were conducted along each transect during a shorter time interval of 1 to 7 days. The first walk on each transect was performed by observer “1” without food delivery and was repeated by observer “2” using food delivery.

To further evaluate the impact of observing FRC through an approach of human intervention (e.g., friendly vocalization and food delivery), on the measured composition of a FRC population, a third observer surveyed the FRC during March to May 2016. These observations were performed on randomly chosen one‐kilometer transects in the residential neighborhoods of the city of Rehovot. Each transect was surveyed twice, back and forth, during the daytime from 6:00 to 8:00 a.m. In the first walk, cats were counted without any intervention; and in the second walk, they were counted with the intervention of friendly calling. The recorded individual variables for each observed cat were similar to those collected in the 2012–2014 part of the study.

### 2.3 Data analysis

#### 2.3.1 Assessing the change in FRC counts over a two‐year follow‐up period

The temporal change in FRC counts was assessed using a generalized linear mixed model (GLMM) with negative binomial distribution. The summary of cat counts in each neighborhood in each year was modeled with the “transect length” set as an offset, “neighborhood” set as the random variable, and “year” as a fixed factor.

#### 2.3.2 Effect of interventional observations on the measured FRC counts

A comparison between two methods should include both correlation and the absolute difference in the measurements acquired by each of the two methods. To determine the correlation of measurements taken according to three observation approaches (no intervention, vocal calling, and vocal calling combined with food delivery), data collected by each of the three observers were analyzed during 2012, 2014, and 2016, separately. Cat counts per km transect and the neutering percentage in each transect walk were calculated. Then, linear regression analysis was performed for the assessed cat counts and neutering percentage for the three observation approaches (no intervention, vocal calling, and vocal calling combined with food delivery). To determine the absolute difference between the three observation approaches, the number of observed cats and percentage of neutered cats were modeled using a GLMM with a Poisson distribution. The “transect” was set as a random effect and “intervention type” (i.e., no intervention vs. friendly calling, and friendly calling vs. vocal calling combined with food delivery) was set as a fixed effect. The “transect length” was set as an offset for the number of observed cats, and the “number of observed cats” was set as an offset for the percentage of neutered cats.

#### 2.3.3 Interobserver and intertransect agreement

Data collected in 2013 were used to determine the interobserver agreement, and a summary of data collected in 2012 and 2014 was analyzed to determine the intertransect agreement in each neighborhood. Cat counts per km transect and the neutering percentage observed in each transect walk were calculated. Linear regression analysis was performed for the assessed cat counts per km and neutering percentage between the two observers and between the two transects in each neighborhood.

#### 2.3.4 Constructive validity of the estimated FRC composition in reference to municipal records

To determine the association of the observed with the expected neutering percentage, municipal records of TNR actions between 2011 and September 2014 were referenced. The geographical location of every cat reported as captured during the TNR campaign was coded, and the number of neutered cats for each neighborhood was calculated. To calculate the ratio of the expected neutered cats, this number was divided by the street lengths in each neighborhood. This ratio was compared to the average proportion of observed neutered FRC in each neighborhood in 2014, using a scatter plot and fitting a linear regression model.

Data were summarized using Microsoft Excel 2016® data spreadsheet. Statistical analysis was performed using lme4 (Bates, Maechler, Bolker, & Walker, 2014) and Car (Fox & Weisberg, 2011) packages in R software (R Core Team, 2014). Unless stated otherwise, in all analyses a significance alpha level of *p* < 0.05 was applied.

## 3 RESULTS

### 3.1 Assessing the change in FRC counts over a two‐year follow‐up period

**Figure 2:**
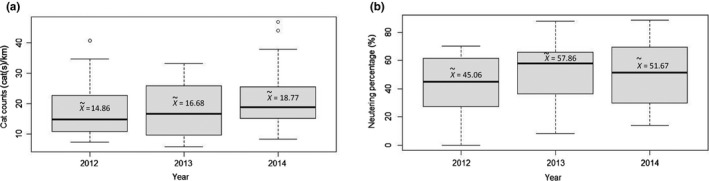
Distribution of the measured cat counts [cats per km] (a) and measured neutering percentage [%] (b) in the neighborhoods of the city of Rishon LeZion (*n* = 27) between the years 2012 and 2014

Overall, 11,733 cat observations were recorded during the current study. The neutering status of these cats was successfully recorded based on the detection of ear cut‐marks, with the exception of 550 (4.69%) individual cat observations due to limited visibility. The annual distribution of cat counts and neutering percentage showed high variability between neighborhoods (a range of 5.8–46.9 cats per km walk, and 0–88.6 neutering percentage, Figure 2). Cat numbers counted in 2014 were significantly higher than in 2012 (prevalence ratio = 1.258, CI_95%_= 1.198–1.322, *p* < 0.001), but did not differ significantly from 2013 (prevalence ratio = 1.032, CI_95%_= 0.982–1.084, *p* = 0.532).

#### 3.1.1 Effect of interventional observations on the measured FRC counts

**Figure 3:**
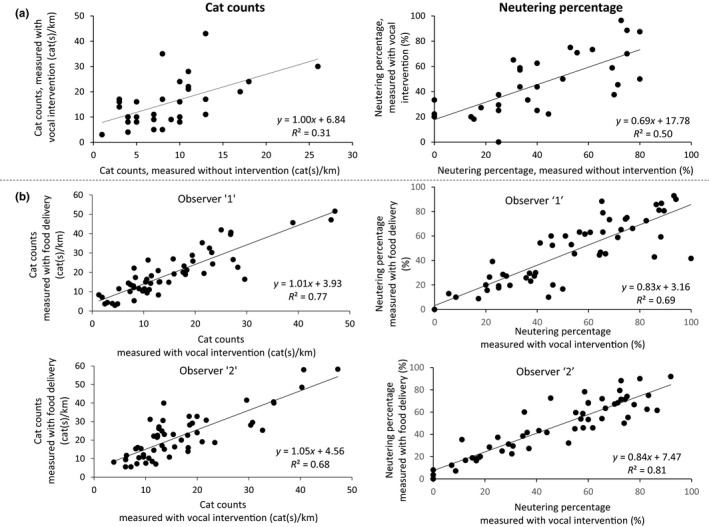
Correlation between cat counts and correlation between estimated percentage of neutered FRCs, with the following interventions: (a) Friendly vocalization versus. no vocalization. Observations were performed twice, back and forth, on each transect during March to May 2016, in the city of Rehovot. (b) Friendly vocalization combined with food delivery versus. vocalization alone (27 transects walked in during 2012 and 2014 [*n* = 54]). Sample surveys were performed by two observers, in two different sets of transects. Each transect walked twice each year, in the city of Rishon LeZion, Israel

Cat counts per km and neutering percentage estimated without any intervention were significantly correlated with the observed values achieved using friendly vocalization (*R* ^2^ = 0.31 and *R* ^2^ = 0.5, *p* = 0.001 and *p* < 0.001, respectively, Figure 3a). Counts of cats and neutering percentage using friendly vocalization were higher by 79% and 18%, respectively (*p* < 0.001 and *p* = 0.131, respectively), compared to the counts of cats observed without any human intervention (Table 2);

**Table 2:** Cat counts and neutering percentage recorded with and without friendly vocalization (calling the cats). Data were collected by one observer during 2016 on *n* = 31 one‐kilometer‐long transects in the city of Rehovot, Israel

Variable	Approach for observation	Cat observations (Mean ± *SD*; Neutered/Total (%))	Ratio between approaches (CI_95%_)	*P*‐value
FRC counts [cat/km]	No intervention	8.7 ± 5.3	1.794 (1.547–2.082)	<.001*
	Friendly calling	15.5 ± 9.4		
Neutering percentage	No intervention	124/268 (46.3%)	1.18 (0.952–1.384)	.131*
	Friendly calling	270/481 (56.1%)		

* Generalized linear mixed model with Poisson distribution

Cat counts and neutering percentage estimated using friendly vocalization were significantly correlated with the values estimated using both vocalization and food delivery (*R* ^2^ = 0.77 and *R* ^2^ = 0.68 for cat counts, and *R* ^2^ = 0.69 and *R* ^2^ = 0.81 for neutering percentage for the two observers, respectively, *p* < 0.001 for all comparisons, Figure 3b). The combination of food delivery with friendly calling resulted in an increase of cat counts per km walk by 30% and by 22% for each observer, respectively (Table 3). Nonetheless, food delivery did not affect the observed percentage of neutered cats (Table 3).

**Table 3:** Cat counts and neutering percentage estimated using friendly vocalization without or in combination with food delivery. Data were collected by two observers during 2012 and 2014 over an overall length of 46.79 km and 54.08 km transect length, respectively, in the city of Rishon LeZion, Israel

Variable	Approach for observation	Cat observations (Observer 1)	Cat observations (Observer 2)
FRC counts [cats/km]	Friendly calling (mean ± sd)	16.1 ± 10.1	18.4 ± 9
	Friendly calling and food delivery (mean ± sd)	20.6 ± 10.8	22.7 ± 11.9
	Mean ratio between approaches (CI_95%_) *p*‐value	1.301 (1.212–1.396) <0.001*	1.219 (1.149–1.294) <0.001*
Neutering percentage [%]	Friendly calling (neutered/total cats)	721/1289 (55.9%) 967/1828 (52.9%)	892/1801 (49.5%) 1084/2235 (48.5%)
	Friendly calling and food delivery (neutered/total cats)		
	Mean ratio between approaches (CI_95%_) *p*‐value	0.947 (0.859–1.043) 0.270*	0.991 (0.907–1.083) 0.839*

* Generalized linear mixed model with Poisson distribution

#### 3.1.2 Interobserver and intertransect agreement

**Figure 4:**
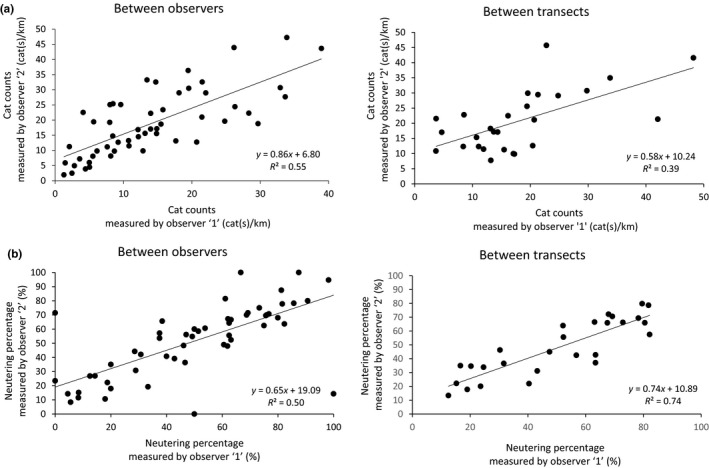
Interobserver and intertransect correlations of cat counts (a) and neutering percentage (b). Surveys were conducted on two transects in each of the 27 neighborhoods in the city of Rishon LeZion, Israel

A high correlation of the estimated cat counts per the same transect was found between the two observers (*R* ^2^ = 0.548, linear regression analysis *p* < 0.001). When each observer walked a different transect, however, the correlation of the average cat counts per km of the two transects was lower (*R* ^2^ = 0.391, linear regression analysis *p* < 0.001, Figure 4a). Moreover, high correlations were found for the neutering percentage both between the two observers (*R* ^2^ = 0.5, linear regression analysis *p* < 0.001) and between the two transects in the same neighborhood (*R* ^2^ = 0.74, linear regression analysis *p* < 0.001, Figure 4b).

#### 3.1.3 Constructive validity of the estimated FRC composition in reference to municipal records

**Figure 5:**
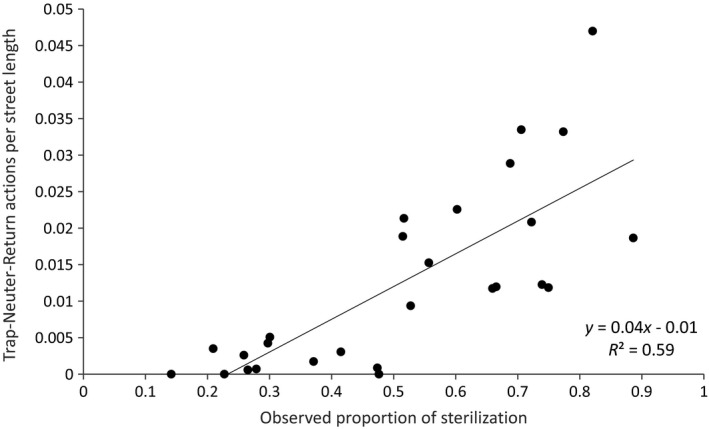
Correlation of the municipal trap–neuter–return actions performed between 2011 and 2014 and the neutering percentage observed in 2014 (27 neighborhoods)

The expected neutering ratio of FRCs according to municipality reports of TNR actions demonstrated a positive high correlation with the observed proportion of neutering (*R* ^2^ = 0.59, linear regression analysis *p* < 0.001). In neighborhoods in which municipal TNR actions were absent, the mean proportion of observed neutering was 0.23 (Figure 5), indicating that additional neutering had been performed independently by citizens.

## 4 DISCUSSION

In the current study, we present an applicable scheme for monitoring FRC metapopulations. Such monitoring is essential, especially in metapopulations, due to the frequent occurrence of changes in population dynamics (e.g., natural emigration or immigration, abandonment or loss of pet cats, adoption of FRCs, and changes in population composition through ongoing TNR actions), all of which affect the abundance, distribution, and density of these populations (Thompson et al., 1998). Answers to questions such as—Has the population increased, decreased, or remained stable; what is the proportion of neutered animals; what is the proportion of young individuals?—are essential when monitoring such populations. In this study, we demonstrate the reliability and constructive validity of an approach using repeated surveys of stratified (neighborhood) random samples (Dohoo, Martin, & Stryhn, 2009; Thompson et al., 1998), in order to answer these questions and, consequently, to demonstrate the applicability of this method for long‐term population monitoring.

The increased cat numbers counted during year 2014 should be interpreted cautiously. According to theoretical models, a constant and high neutering rate of above 70% is required for inducing a reduction in cat population (Foley, Foley, Levy, & Paik, 2005; McCarthy, Levine, & Reed, 2013). Such a reduction is expected only after exceeding the expected lag‐time effect of neutering (Boone, 2015; Natoli et al., 2006). The current study does not meet these two requirements for assessing TNR efficacy. The determination of population trend can be analyzed by a regression model, as presented here. However, for the nonprofessional staff, a quick and simpler alternative of the nonparametric Mann–Kendall ranking procedure test can be used. This latter test has the advantage of assessing trends without requiring the use of exact estimates of population size. Rather, it only requires a minimum of four observational periods (not necessarily consecutive) and the ability to rank the observations over years. For detailed explanation of this analysis, see Thompson et al. (1998).

Ecologists often use indirect indices for estimating the population size of small‐sized wildlife mammals, such as nesting or resting structures, vocalizations, feces, and hair of the counted animals (Krebs, 2006), or the more recent method of direct observations achieved by camera traps, which often use bait to attract the target animals (Rowcliffe, Field, Turvey, & Carbone, 2008). Such approaches are mostly used to circumvent the avoidance behavior of these mammals. Direct counts are nonetheless preferred over indirect indices, and collecting data pertaining to every individual in the sample unit is desirable (Greenwood & Robinson, 2006; Thompson et al., 1998). Living in an urban setting, both free‐roaming domestic species and wild species frequently encounter humans. As a result, these animals might present with less timid behavior than feral and wild mammals that live in natural habitats. Taking into account this expected behavioral adjustment of FRC to humans, we were able to enhance the effectiveness of direct observations by either drawing the cats out using specific friendly vocalizations or by means of food delivery. These interventions can be regarded as a specific modification of bait. This approach was taken in an attempt to increase the detectability (visibility) of FRC in the sampled unit (Greenwood & Robinson, 2006; Dohoo et al., 2009), overcoming the difficulties in sampling a small‐body mammal with a wealth of access to hiding places.

Interventional observation using friendly vocalization resulted in significantly higher counts per km (by 79%) of detected FRC than for noninterventional observation, but did not alter the estimated percentage of neutered cats. These findings expand those of Kilgour et al. (2017), who demonstrated that sightability (observing without intervention) is not affected by sterilization. Observations accompanied by food delivery were associated with a further higher number of detected cats (24%–28%), compared to the use of vocalization alone, but, again, this did not alter the estimated percentage of neutered cats.

During the observations, some of the cats reacted to vocalization by running toward the observers in the anticipation of food. When food was not delivered, the cats returned to their hiding places shortly afterward. When food was delivered, the cats remained nearby the observer in order to eat. Within a few minutes after the first cat had begun eating, other cats joined in. As a result, delivering food resulted not just in an increase in cat detectability, but it also provided closer observation of the cats, which is an advantage when estimating their neutering status and age.

Cat detectability is influenced by both the cats' behavior and that of the observer, such as the intensity of the friendly vocalization and the walking pace. Moreover, transient changes in the surroundings can also affect cat detectability, especially changes in human activity that might lead to the cats fleeing or hiding. Taking into account such differences and the few days' interval between observations, the interobserver correlations indicate a good agreement. However, in order to reduce the variability, it is advised to conduct observations by as few observers as possible.

In contrast to the interobserver agreement that is affected by temporal dynamics, the intertransect agreement (of the same neighborhood) is affected by the spatio‐temporal dynamics. Thus, it could be expected that the intertransect agreement would be low. Nonetheless, the intertransect agreement was fair for cat counts and very good for neutering percentage. Consequently, it is possible that the measurement of cat counts is more sensitive to environmental changes than the measurement of neutering percentage.

The results of the intertransect agreement and its correlation with municipal TNR actions indicate a good reliability and validity of the estimated neutering percentage. The importance of this finding lies in the goal of TNR programs, which is to reduce FRC populations. In order to achieve this goal, maintaining a minimum of 75% sterilization should be achieved and monitored throughout the years (McCarthy et al., 2013, Andersen, Martin, & Roemer, 2004, Budke & Slater, 2009, Miller et al., 2014, Boone, 2015).

The generalization of the efficacy of the proposed scheme for monitoring other FRC populations or other domesticated or wildlife populations should be considered in light of the animals' feeding resources and their habituation to humans. Populations that are habituated to deliberated feeding by humans have greater potential to be monitored using the proposed scheme. Such populations may be for example: FRC in Malaysia (Khor, Davey, & Zhao, 2018), in the United States (Cove, Gardner, Simons, Kays, & O'Connell, 2018; Levy, Woods, Turick, & Etheridge, 2003), in Italy (Natoli et al., 2006; Slater et al., 2008), in Japan (Seo & Tanida, 2018), and in Greece (Mannhart, 2007); free‐roaming dogs in Nepal (Massei et al., 2017), in Greece (Mannhart, 2007), and in Italy (Slater et al., 2008); and feral pigeons and various bird species throughout the western world (Jones and James Reynolds, 2008, Belguermi et al.., 2011).

One of the current study's limitations was that of sighting distance, which depends on the urban architecture around the transects, which changes between and along the transects. As a result, the sampling area is not constant, hindering calculation of the mean and variance of cat densities per square km in the city. As noted above, although such calculation is very interesting, it is not a prerequisite for monitoring control strategies over time. Other study limitations include documenting the same cat more than once and only partial documentation of cats during each observational walk. Double documentation of the same cat depends on the observer's experience and can be avoided by recording other individual characteristics as was done in Gunther et al. (2018). Partial documentation depends more on cat behavior and might lead to an underestimate of cat counts. We believe that partial documentation is more common and could be reduced by friendly calling the cats, delivering them palatable food, and also walking at a slower pace.

In summary, we present here an applicable monitoring scheme for FRC metapopulations in the urban environment. While this scheme does not enable determination of the absolute population size, its high reliability and repeatability are suitable for uncovering trends regarding the observed population size and proportion of managed individuals. The design of such a monitoring scheme should include first the determination of the area and period of interest. Then, a geographical stratification and the time interval between sampling should be determined. For maintaining consistency, it is advised to perform observations each year at the same season. The use of friendly vocalization in combination with food delivery improves cat detectability and contributes to the accuracy of the scheme. Though moderate correlation was documented between observers, we recommend that observations will be performed by as few observers as possible. Assuming that the detectability of FRC does not change over the years, this scheme could be used for data collection without the need to mark or individually identify large number of FRCs in large areas.

## CONFLICT OF INTEREST

None.

## AUTHORS CONTRIBUTION

Gunther I. contributed to conception and design, acquisition of data, analysis and interpretation of data, and drafting and revising the manuscript; Azriel L. and Wolf H. contributed to acquisition of data; Raz T. contributed to conception and design, and revising the manuscript; Klement E. contributed to conception and design, analysis and interpretation of data, and revising the manuscript.

### Open Research Badges

This article has earned an Open Materials Badge for making publicly available the components of the research methodology needed to reproduce the reported procedure and analysis. All materials are available at http://doi.org/10.5281/zenodo.3572833.

## Data Availability

Data and R scripts are available at Zenodo: http://doi.org/10.5281/zenodo.3572833

## References

[ece35982-bib-0001] Andersen, M. C. , Martin, B. J. , & Roemer, G. W. (2004). Use of matrix population models to estimate the efficacy of euthanasia versus trap‐neuter‐return for management of free‐roaming cats. Journal of the American Veterinary Medical Association, 225, 1871–1876. 15643836 10.2460/javma.2004.225.1871

[ece35982-bib-0002] Bates, D. , Maechler, M. , Bolker, B. , & Walker, S. (2014). lme4: Linear mixed‐effects models using Eigen and S4. R Package Version, 1, 1–23.

[ece35982-bib-0003] Belguermi, A. , Bovet, D. , Pascal, A. , Prévot‐Julliard, A.‐C. , Saint Jalme, M. , Rat‐Fischer, L. , & Leboucher, G.. (2011). Pigeons discriminate between human feeders. Animal Cognition, 14, 909. 21647649 10.1007/s10071-011-0420-7

[ece35982-bib-0004] Belo, V. S. , Werneck, G. L. , da Silva, E. S. , Barbosa, D. S. , & Struchiner, C. J. (2015). Population estimation methods for free‐ranging dogs: A systematic review. PLoS ONE, 10, e0144830. 26673165 10.1371/journal.pone.0144830 PMC4684217

[ece35982-bib-0005] Boone, J. D. (2015). Better trap–neuter–return for free‐roaming cats using models and monitoring to improve population management. Journal of Feline Medicine and Surgery, 17, 800–807. 26323805 10.1177/1098612X15594995 PMC11148983

[ece35982-bib-0006] Boone, J. D. , Miller, P. , Briggs, J. R. , Benka, V. A. , Lawler, D. F. , Slater, M. R. , … Zawistowski, S. (2019). A long‐term lens: Cumulative impacts of free‐roaming cat management strategy and intensity on preventable cat mortalities. Frontiers in Veterinary Science, 6, 238. 31403048 10.3389/fvets.2019.00238 PMC6676151

[ece35982-bib-0007] Boone, J. D. , & Slater, M. (2014).Counting cats: Recommendations for population monitoring programs to inform the management of free‐roaming cats.[Accessed 26 October 2016].

[ece35982-bib-0008] Budke, C. M. , & Slater, M. R. (2009). Utilization of matrix population models to assess a 3‐year single treatment nonsurgical contraception program versus surgical sterilization in feral cat populations. Journal of Applied Animal Welfare Science, 12, 277–292. 20183481 10.1080/10888700903163419

[ece35982-bib-0009] Calhoon, R. E. , & Haspel, C. (1989). Urban cat populations compared by season, subhabitat and supplemental feeding. The Journal of Animal Ecology, 58, 321–328.

[ece35982-bib-0010] Cove, M. V. , Gardner, B. , Simons, T. R. , Kays, R. , & O'Connell, A. F. (2018). Free‐ranging domestic cats (Felis catus) on public lands: Estimating density, activity, and diet in the Florida Keys. Biological Invasions, 20, 333–344. 10.1007/s10530-017-1534-x

[ece35982-bib-0011] Cuffe, D. , Eachus, J. , Jackson, O. , Neville, P. , & Remfry, J. (1983). Ear‐tipping for identification of neutered feral cats. Veterinary Record, 112(6), 129. 6836893 10.1136/vr.112.6.129

[ece35982-bib-0012] Denny, E. A. , & Dickman, C. (2010). Review of cat ecology and management strategies in Australia. Canberra: Invasive Animals Cooperative Research Centre.

[ece35982-bib-0013] Devillard, S. , Say, L. , & Pontier, D. (2003). Dispersal pattern of domestic cats (Felis catus) in a promiscuous urban population: Do females disperse or die? Journal of Animal Ecology, 72, 203–211.

[ece35982-bib-0014] Dohoo, I. R. , Martin, W. , & Stryhn, H. (2009). Ch. 2: Sampling In: Veterinary epidemiologic research *.* (2nd edition ed.). Charlottetown, Canada: AVC Incorporated.

[ece35982-bib-0015] Finkler, H. , Hatna, E. , & Terkel, J. (2011). The influence of neighbourhood socio‐demographic factors on densities of free‐roaming cat populations in an urban ecosystem in Israel. Wildlife Research, 38, 235–243. 10.1071/WR10215

[ece35982-bib-0016] Flockhart, D. , Norris, D. , & Coe, J. (2016). Predicting free‐roaming cat population densities in urban areas. Animal Conservation, 19, 472–482.

[ece35982-bib-0017] Foley, P. , Foley, J. E. , Levy, J. K. , & Paik, T. (2005). Analysis of the impact of trap‐neuter‐return programs on populations of feral cats. Journal of the American Veterinary Medical Association, 227, 1775–1781. 16342526 10.2460/javma.2005.227.1775

[ece35982-bib-0018] Fox, J. , & Weisberg, S. (2011). Multivariate linear models in R In: An R companion to applied regression. 2 Los Angeles, Thousand Oaks: SAGE Publications.

[ece35982-bib-0019] Galvis, J. O. A. , Baquero, O. S. , Dias, R. A. , Ferreira, F. , Chiozzotto, E. N. , & Grisi‐Filho, J. H. H. . (2015). Monitoring techniques in the capture and adoption of dogs and cats. Geospatial Health, 10, 158-162. 10.4081/gh.2015.339 26618312

[ece35982-bib-0020] Gerhold, R. W. , & Jessup, D. A. (2013). Zoonotic diseases associated with free‐roaming cats. Zoonoses and Public Health, 60, 189–195. 22830565 10.1111/j.1863-2378.2012.01522.x

[ece35982-bib-0021] Greenwood, J. J. D. , & Robinson, R.A. (2006). Principles of sampling In:Sutherland W. J.(Ed.), Ecological census techniques: A handbook (pp 11-86). Cambridge, UK: Cambridge University Press.

[ece35982-bib-0023] Gunther, I. , Finkler, H. , & Terkel, J. (2011). Demographic differences between urban feeding groups of neutered and sexually intact free‐roaming cats following a trap‐neuter‐return procedure. Javma‐Journal of the American Veterinary Medical Association, 238, 1134–1140. 10.2460/javma.238.9.1134 21529235

[ece35982-bib-0024] Gunther, I. , Raz, T. , Berke, O. , & Klement, E. (2015). Nuisances and welfare of free‐roaming cats in urban settings and their association with cat reproduction. Preventive Veterinary Medicine, 119, 203–210. 25770734 10.1016/j.prevetmed.2015.02.012

[ece35982-bib-0025] Gunther, I. , Raz, T. , & Klement, E. (2018). Association of neutering with health and welfare of urban free‐roaming cat population in Israel, during 2012–2014. Preventive Veterinary Medicine, 157, 26–33. 10.1016/j.prevetmed.2018.05.018 30086846

[ece35982-bib-0026] Hiby, L. R. , Reece, J. F. , Wright, R. , Jaisinghani, R. , Singh, B. , & Hiby, E. F. (2011). A mark‐resight survey method to estimate the roaming dog population in three cities in Rajasthan. India. BMC Veterinary Research, 7, 46. 21834979 10.1186/1746-6148-7-46 PMC3163189

[ece35982-bib-0027] Hughes, K. L. , Slater, M. R. , & Haller, L. (2002). The effects of implementing a feral cat spay/neuter program in a Florida county animal control service. Journal of Applied Animal Welfare Science, 5, 285–298. 16221079 10.1207/S15327604JAWS0504_03

[ece35982-bib-0028] Izawa, M. , Doi, T. , & Ono, Y. (1991). Notes on the spacing pattern of the feral cats at high density. Bulletin Kitakyushu Museum of Natural History, 10, 109–113.

[ece35982-bib-0029] Jones, A. L. , & Downs, C. T. (2011). Managing feral cats on a university's campuses: How many are there and is sterilization having an effect? Journal of Applied Animal Welfare Science, 14, 304–320. 21932945 10.1080/10888705.2011.600186

[ece35982-bib-0030] Jones, D. N. , & James Reynolds, S. (2008). Feeding birds in our towns and cities: A global research opportunity. Journal of Avian Biology, 39, 265–271.

[ece35982-bib-0031] Kaeuffer, R. , Pontier, D. , Devillard, S. , & Perrin, N. (2004). Effective size of two feral domestic cat populations (*Felis* *catus* L.): Effect of the mating system. Molecular Ecology, 13, 483–490. 14717902 10.1046/j.1365-294x.2003.02046.x

[ece35982-bib-0032] Khor, M. M. , Davey, G. , & Zhao, X. (2018). Why do people feed free‐roaming cats? The role of anticipated regret in an extended theory of planned behavior in Malaysia. Anthrozoös, 31, 101–116. 10.1080/08927936.2018.1406204

[ece35982-bib-0033] Kilgour, R. , Magle, S. , Slater, M. , Christian, A. , Weiss, E. , & Ditullio, M. (2017). Estimating free‐roaming cat populations and the effects of one year Trap‐Neuter‐Return management effort in a highly urban area. Urban Ecosystems, 20, 207–216. 10.1007/s11252-016-0583-8

[ece35982-bib-0034] Krebs, C. (2006). Mammals In: Sutherland W. J. (Ed.) Ecological Census Techniques, a Handbook, (2nd). pp: 351-369. Cambridge, UK: Cambridge University Press.

[ece35982-bib-0035] Kreisler, R. E. , Cornell, H. N. , & Levy, J. K. (2019). Decrease in population and increase in welfare of community cats in a twenty‐three year trap‐neuter‐return program in Key Largo, FL: The ORCAT program. Frontiers in Veterinary Science, 6, 1-14. 30775368 10.3389/fvets.2019.00007 PMC6367225

[ece35982-bib-0036] Lepczyk, C. A. , Dauphiné, N. , Bird, D. M. , Conant, S. , Cooper, R. J. , Duffy, D. C. , … Temple, S. A. (2010). What conservation biologists can do to counter trap‐neuter‐return: Response to Longcore et al Conservation Biology, 24(2), 627–629. 10.1111/j.1523-1739.2009.01426.x 20088960

[ece35982-bib-0037] Levy, J. K. , Woods, J. E. , Turick, S. L. , & Etheridge, D. L. (2003). Number of unowned free‐roaming cats in a college community in the southern United States and characteristics of community residents who feed them. Journal of the American Veterinary Medical Association, 223, 202–205. 12875446 10.2460/javma.2003.223.202

[ece35982-bib-0038] Longcore, T. , Rich, C. , & Sullivan, L. M. (2009). Critical assessment of claims regarding management of feral cats by trap‐neuter‐return. Conservation Biology, 23, 887–894. 19245489 10.1111/j.1523-1739.2009.01174.x

[ece35982-bib-0039] Loss, S. R. , & Marra, P. P. (2018). Merchants of doubt in the free‐ranging cat conflict. Conservation Biology: the Journal of the Society for Conservation Biology, 32(2), 265–266. 29377342 10.1111/cobi.13085

[ece35982-bib-0040] Loss, S. R. , Will, T. , & Marra, P. P. (2013). The impact of free‐ranging domestic cats on wildlife of the United States. Nature Communications, 4, 1396. 10.1038/ncomms2380 23360987

[ece35982-bib-0041] Mannhart, T. (2007). A catch‐neuter‐release project for free‐roaming dogs and cats in Rhodes, Greece: Problem analysis and effectiveness of the strategy. PhD thesis, University of Bern.

[ece35982-bib-0042] Massei, G. , Fooks, A. , Horton, D. , Callaby, R. , Sharma, K. , Dhakal, I. , & Dahal, U. (2017). Free‐roaming dogs in Nepal: Demographics, health and public knowledge, attitudes and practices. Zoonoses and Public Health, 64, 29–40. 27334892 10.1111/zph.12280

[ece35982-bib-0043] McCarthy, R. J. , Levine, S. H. , & Reed, J. M. (2013). Estimation of effectiveness of three methods of feral cat population control by use of a simulation model. Journal of the American Veterinary Medical Association, 243, 502–511. 23902443 10.2460/javma.243.4.502

[ece35982-bib-0044] Miller, P. S. , Boone, J. D. , Briggs, J. R. , Lawler, D. F. , Levy, J. K. , Nutter, F. B. , … Zawistowski, S. (2014). Simulating free‐roaming cat population management options in open demographic environments. PLoS ONE, 9(11), e113553 25426960 10.1371/journal.pone.0113553 PMC4245120

[ece35982-bib-0045] Mirmovitch, V. (1995). Spatial organisation of urban feral cats (Felis catus) in Jerusalem. Wildlife Research, 22, 299–310.

[ece35982-bib-0046] Morters, M. K. , Restif, O. , Hampson, K. , Cleaveland, S. , Wood, J. L. , & Conlan, A. J. (2013). Evidence‐based control of canine rabies: A critical review of population density reduction. Journal of Animal Ecology, 82, 6–14. 23004351 10.1111/j.1365-2656.2012.02033.x PMC3579231

[ece35982-bib-0047] Moseby, K. , Peacock, D. , & Read, J. (2015). Catastrophic cat predation: A call for predator profiling in wildlife protection programs. Biological Conservation, 191, 331–340.

[ece35982-bib-0048] Natoli, E. (1985). Spacing pattern in a colony of urban stray cats (*Felis* *catus* L.) in the historic centre of Rome. Applied Animal Behaviour Science, 14, 289–304.

[ece35982-bib-0049] Natoli, E. , Maragliano, L. , Cariola, G. , Faini, A. , Bonanni, R. , Cafazzo, S. , & Fantini, C. (2006). Management of feral domestic cats in the urban environment of Rome (Italy). Preventive Veterinary Medicine, 77, 180–185. 17034887 10.1016/j.prevetmed.2006.06.005

[ece35982-bib-0050] Nutter, F. B. , Levine, J. F. , & Stoskopf, M. K. (2004). Reproductive capacity of free‐roaming domestic cats and kitten survival rate. Journal of the American Veterinary Medical Association, 225, 1399–1402. 15552315 10.2460/javma.2004.225.1399

[ece35982-bib-0051] Page, R. , Ross, J. , & Bennet, D. (1992). A study of the home ranges, movements and behaviour of the feral cat population at Avonmouth Docks. Wildlife Research, 19, 263–277.

[ece35982-bib-0052] Peterson, M. N. , Hartis, B. , Rodriguez, S. , Green, M. , & Lepczyk, C. A. (2012). Opinions from the front lines of cat colony management conflict. PLoS ONE, 7, e44616. 22970269 10.1371/journal.pone.0044616 PMC3435309

[ece35982-bib-0053] Punjabi, G. A. , Athreya, V. , & Linnell, J. D. (2012). Using natural marks to estimate free‐ranging dog Canis familiaris abundance in a Mark‐Resight framework in suburban Mumbai, India. Tropical Conservation Science, 5, 510–520.

[ece35982-bib-0054] R Core Team . (2014). R: A language and environment for statistical computing. Vienna, Austria: R Foundation for Statistical Computing.

[ece35982-bib-0055] Rowcliffe, J. M. , Field, J. , Turvey, S. T. , & Carbone, C. (2008). Estimating animal density using camera traps without the need for individual recognition. Journal of Applied Ecology, 45, 1228–1236.

[ece35982-bib-0056] Say, L. , Pontier, D. , & Natoli, E. (1999). High variation in multiple paternity of domestic cats (*Felis* *catus* L.) in relation to environmental conditions. Proceedings of the Royal Society of London B: Biological Sciences, 266, 2071–2074. 10.1098/rspb.1999.0889 PMC1690320 10902544

[ece35982-bib-0057] Schmidt, P. M. , Pierce, B. L. , & Lopez, R. R. (2007). Estimating free‐roaming cat densities in urban areas: Comparison of Mark‐Resight and distance sampling. Wildlife Biological Practice, 3, 18–27.

[ece35982-bib-0058] Seo, A. , & Tanida, H. (2018). Three‐year route census study on welfare status of free‐roaming cats in old‐town Onomichi, Japan. Journal of Applied Animal Welfare Science, 21, 203–210. 28960090 10.1080/10888705.2017.1379401

[ece35982-bib-0059] Slater, M. R. , di Nardo, A. , Pediconi, O. , Villa, P. D. , Candeloro, L. , Alessandrini, B. , & del Papa, S. (2008). Free‐roaming dogs and cats in central Italy: Public perceptions of the problem. Preventive Veterinary Medicine, 84, 27–47. 18055046 10.1016/j.prevetmed.2007.10.002

[ece35982-bib-0060] Thompson, W. L. , White, G. C. , & Gowan, C. (1998). Monitoring vertebrate populations. San Diego, California, USA: Academic Press.

[ece35982-bib-0061] Zito, S. , Aguilar, G. , Vigeant, S. , & Dale, A. (2018). Assessment of a targeted trap‐neuter‐return pilot study in Auckland. New Zealand. Animals, 8, 73. 10.3390/ani8050073 PMC5981284 29757255

